# Genome-Wide Identification, Expression Profiling, and Characterization of Cyclin-like Genes Reveal Their Role in the Fertility of the Diamondback Moth

**DOI:** 10.3390/biology11101493

**Published:** 2022-10-12

**Authors:** Muhammad Asad, Jing Chen, Jianying Liao, Dan Liu, Jiajing Yu, Guang Yang

**Affiliations:** 1 State Key Laboratory of Ecological Pest Control for Fujian and Taiwan Crops, Institute of Applied Ecology, Fujian Agriculture and Forestry University, Fuzhou 350002, China; 2 Joint International Research Laboratory of Ecological Pest Control, Ministry of Education, Fuzhou 350002, China; 3 Key Laboratory of Integrated Pest Management for Fujian-Taiwan Crops, Ministry of Agriculture, Fuzhou 350002, China; 4 Key Laboratory of Green Pest Control, Fujian Province University, Fuzhou 350002, China; 5 Ministerial and Provincial Joint Innovation Centre for Safety Production of Cross Strait Crops, Fujian Agriculture and Forestry University, Fuzhou 350002, China

**Keywords:** *Plutella xylostella*, cyclin-like gene family, phylogenetic relation, gene structure, expression pattern, *Cis*-regulatory element, micro-RNA, RNA interference

## Abstract

**Simple Summary:**

Cyclin genes are known as cell cycle regulators and play a significant role in the fertility of different organisms, including mice and insects. Until now, no study has been performed on the complete identification of the cyclin genes in insects. Here, we identified 21 cyclin genes in the diamondback moth (DBM) genome through a comprehensive genome-wide analysis and evaluated the gene structure, genomic location, and evolutionary relationship. *Cis*-regulatory elements and potential miRNA targeting the cyclin genes were also assessed. By analyzing the transcriptomic and RT-qPCR based expression profiling at different stages and tissues, we found that the majority of the cyclin genes were significantly expressed in the reproductive tissues. Moreover, RNAi-mediated characterization of *PxCyc B1* showed its role in female fertility. The current study provides a basis for further evaluation of the cyclin genes, which may be used as a potential target for pest management programs.

**Abstract:**

Cyclin-like genes are primarily considered as cell cycle regulators and have shown to be crucial for insect growth, development, reproduction, and fertility. However, no research has been performed on the cyclin-like genes in the diamondback moth (*Plutella xylostella*). Here, we identified the 21 cyclin genes in the genome of *P. xylostella* and clustered them into four groups. Most cyclin genes showed a well-maintained gene structure and motif distribution within the same group. The putative promoter regions of cyclin genes contained several transcription binding factors related to reproduction, along with growth and development. Furthermore, 16 miRNAs were identified targeting the 13 cyclin genes. Transcriptome and quantitative real-time PCR (qRT-PCR)-based expression profiling of cyclin-like genes at different stages and tissues were evaluated, revealing that 16 out of 21 cyclin genes were highly expressed in reproductive tissues of adult females and males. The Cyclin B1 gene (*PxCyc B1*) was only expressed in the ovary of the adult female and selected for the subsequent analysis. RNAi-mediated suppression of *PxCyc B1* interrupted the external genitalia and length of the ovariole of female adults. Furthermore, the egg-laying capacity and hatching rate were also significantly decreased by suppressing the *PxCyc B1*, indicating the importance of cyclin genes in the reproduction and fertility of *P. xylostella*. The current study explained the detailed genome-wide analysis of cyclin-like genes in *P. xylostella*, which provided a basis for subsequent research to assess the roles of cyclin genes in reproduction, and the cyclin gene may be considered an effective target site to control this pest.

## 1. Introduction

The most important and fundamental aspect of biological growth and development is cell division, and any consideration of the significance of cell division in insect growth, development, and reproduction demands a comprehensive knowledge of primary mechanisms that control the cell cycle [1]. The G1-S-G2-M transition in the cell cycle is primarily regulated by the enzymatic activities of cyclin-dependent kinases (CDKs). CDK enzymatic activities are controlled through complex ways, such as cyclin activation and binding; binding of the CDK subunit (CKS) and inhibitor protein of CDK (CKI); CDK dephosphorylation/phosphorylation; intracellular tracking; proteolysis; E2F transcription factors (E2F); homologs of retinoblastoma protein (Rb); and the dimerization partner (DP) pathway [2]. Cyclins and CDKs are the most critical cell cycle regulator proteins among these protein factors [2]. The interaction of cyclins with CDKs is required to activate these complexes [3,4]. Changes in cyclin concentration produce sequential activation or deactivation of the CDK catalytic partners, leading to the periodic development of cells through the cell cycle [5,6]. Following the discovery of the cyclin protein in sea urchin oocytes [7], more cyclins, CKIs, CDKs, and E2F transcription factors were identified in both plants and mammals [4,8,9]. Different cyclin-like genes were fully characterized, and a few cyclins (cyclins A, B, D, and E) seem to be key players in cell cycle regulation and reproduction [10]. However, other cyclins, including cyclins C, K, H, and T, participate in transcriptional activities [11,12]. The typical cyclin has a conserved region known as a cyclin core and comprises two domains, cyclin C and cyclin N [13]. Cyclin N is a highly conserved domain recognized as the cyclin box, whereas cyclin C is less conserved. Various cyclin-related proteins only contain the cyclin N domain without the cyclin C domain [14], indicating that the function of the cyclin C domain might not be required.

In *Drosophila melanogaster*, cyclin A and B are expressed together in entire cell phases. However, cyclin A is sufficient for mitosis, and cyclin B is not required for mitosis [15]. Cyclins A, B, and B3 together regulate mitosis, but cyclins B and B3 are essential for male and female fertility in *D. melanogaster* [16]. Cyclin G plays a vital role in the wing development of *D. melanogaster*. Both overexpression and RNAi-mediated suppression of *DmCyc G* change the symmetry of wings, indicating DmCyc G’s role in wing developmental stability in *D. melanogaster* [12]. Cyclin C and CDK8 are closely linked to nutritional intake and fat metabolism during larva to pupa transitions in *D. melanogaster* [17]. Furthermore, the DmCyc G negatively regulates the cell growth and development process since overproduction of DmCyc G results in a limited number of cells, while a lack of DmCyc G results in a large number of cells [18]. In *Bombyx mori*, the suppression of *BmCyclin B* and *BmCyclin B3* expression through RNAi causes cell cycle detention at the G2 and M phases in the BmN cell line, reducing cell proliferation, which indicates that BmCyclin B and BmCyclin B3 are required for completion of the cell cycle in the BmN cell line [19]. Previous reports in *D. melanogaster* and *B. mori* suggest that cyclin-like genes are not only involved in cell cycle regulations but play significant roles in growth, development, and reproduction.

The diamondback moth (*Plutella xylostella*, Lepidoptera: Plutellidae) is a highly destructive pest worldwide and is considered the primary agent responsible for damaging cruciferous crops because of its short life span, rapid reproduction, high feeding rate at the larval stage, high genetic diversity, and distribution [20,21]. Currently, the management of *P. xylostella* is accomplished by applying different pesticides harmful to humans, natural enemies, and the environment. Moreover, *P. xylostella* has developed resistance against major pesticides due to its rapid overlapping generations [22,23]. To maintain this pest, new and innovative approaches are urgently required, such as the identification of a potential target site for a CRISPR/Cas9-based gene-drive system [24]. The identification and characterizations of genes involved in fertility may be used as target sites to manage this pest. Previously, it has been reported that some cyclin genes are responsible for male and female fertility in *D. melanogaster* [16]. Although several genes in the *P. xylostella* genome were annotated as cyclin-like genes [25]; however, no study was conducted about the complete identification of the cyclin-like gene family in *P. xylostella*. Here, we identified the 21 cyclin-like genes in *P. xylostella*. Furthermore, we analyzed the phylogenetic relationship, gene structure, expression profile, and role in the fertility of *P. xylostella*.

## 2. Materials and Methods

### 2.1. Rearing of Plutella xylostella

The artificial diet strain Geneva 88 (G88) of *P. xylostella* was used, which was obtained from Cornell University and established at the Institute of Applied Ecology, Fujian Agriculture and Forestry University. The artificial diet was prepared by mixing different components such as agar, linoleic acid, canola oil, sucrose, radish seed powder, methyl paraben, vitamin, potassium sorbate ascorbic acid, and raw wheat material. The detailed procedure of the artificial diet preparation was described previously [26,27]. The newly hatched larvae of *P. xylostella* were maintained on an artificial diet until they developed into pupae at 25 °C, 35–50% relative humidity, and 16 h:8 h (light:dark) photoperiod. The pupae were collected and transferred into the mating chamber. The 10% honey solution was used to feed the adults.

### 2.2. Identification of Putative Cyclin-like Genes

Two methods were used to identify the cyclin-like genes from the *P. xylostella* genome. The amino acid sequences of cyclin-like genes of *D. melanogaster* and *Bombyx mori* were downloaded from NCBI (https://www.ncbi.nlm.nih.gov// accessed on 10 May 2022). The obtained sequences were used as a query to blast against the *P. xylostella* transcriptome by using the BLASTX algorithm with 10^−5^ threshold E-value. The second method to identify the remaining cyclin-like genes was conducted by using HMMER3 software. The cyclin N-terminal and C-terminal domain files were downloaded from PFAM (http://pfam.janelia.org/ accessed on 10 May 2022). These obtained files were used as a query against the *P. xylostella* protein dataset to search cyclin-like genes using the hmmscan program of Tbtool software [28]. The best-matched cyclin-like candidate genes were selected to confirm the conserved domain by using the online motif search tool. The genes without containing conserved domains were manually removed from the list.

### 2.3. Gene Structure and Motif Analysis

The genomic DNA sequences and cDNA sequences of *P. xylostella* cyclins were downloaded from the *P. xylostella* database; then, these sequences were analyzed by using the online available Gene Structure Display Server tool [28]. To identify the motif structure of cyclin genes, the protein sequences of *P. xylostella* cyclins were merged with the help of the GENESTUDIO program (http://www.genestudio.com/ accessed on 12 May 2022). Then, these merged protein sequences were further analyzed by using the MEME program (https://meme-suite.org/meme/tools/meme accessed on 12 May 2022). The schematic diagram of gene structure and conserved motif was prepared by using the Tbtool software [29].

### 2.4. Genomic Location and Phylogenetic Analysis

To determine the genomic location of cyclin genes, the general feature format (GFF) file was obtained from the *P. xylostella* genomic database (http://59.79.254.1/DBM/ accessed on 15 May 2022) [30]. The locations of 21 cyclin genes on the different scaffolds were identified by using the TBtools software [29]. To study the evolutionary relationship, the amino acid sequences of cyclin-like genes of three representative insect species belonging to three different orders were obtained and aligned with the cyclin-like genes of *P. xylostella* by using the CLUSTALW. The phylogenetic tree was constructed from these multiple aligned protein sequences by using the MEGA10 software with bootstrap value 1000 [31].

### 2.5. Physiochemical Properties of Cyclin Genes

Subcellular localization of the cyclin gene in a different part of the cells was predicted by using the Cell-PLoc 2.0 (http://www.csbio.sjtu.edu.cn/bioinf/Cell-PLoc-2/ accessed on 15 May 2022) [32], and other physiochemical properties, such as theoretical isoelectric point (PI) and molecular weight (MW) were predicted by the Expasy server [33].

### 2.6. Cis-Regulatory Elements Analysis

For *cis*-regulatory elements analysis of cyclin gene, the 2-kb upstream sequences of cyclin genes were obtained from the genome and scanned at the ALGGEN server: PROMO and MALGEN [34]. Acquired binding sites were analyzed manually and selected based on their functions in insects. The schematic representation of *cis*-regulatory elements was prepared by using the TBtools software [29].

### 2.7. Prediction of miRNAs Targeting the Cyclin Genes of P. xylostella

The coding sequences of cyclin genes were obtained and used for the prediction of putative miRNAs targeting the cyclin genes. The miRNA sequences were obtained from the Insectbase 2.0 (http://v2.insect-genome.com/ accessed on 16 May 2022) [35]. Acquired miRNA and coding sequences of cyclin-like genes were scanned at the RNA hybrid BiBi server by using the default parameter (https://bibiserv.cebitec.uni-bielefeld.de/rnahybrid/ accessed on 16 May 2022) [36]. The schematic diagram of the network interaction miRNAs targeting the cyclin gene was constructed by using the Cytoscape software version 3.8.2 [37].

### 2.8. Stage- and Tissue-Specific Expression Profiling of Cyclin Genes

Previously, we performed transcriptome-based expression profiling at different stages and tissues in *P. xylostella* [25]. These transcriptomic data were used to study the stage- and tissue-specific expressions of cyclin genes. The expressions of cyclin genes in fragments per kilobase million (FPKM) were observed in various stages and tissues. The obtained FPKM were normalized with log2 and used to develop heatmaps at different stages and tissues by using the TBtools software [29].

### 2.9. RNA Extraction, cDNA Preparation and RT-qPCR Analysis

To evaluate the relative expression levels of cyclin genes, the total RNA was extracted from different stages (eggs, first-instar larvae, second-instar larvae, third-instar larvae, fourth-instar male and female larvae, male and female pupae, and male and female adults) and tissues (head, integument, fat body, malpighian tubule, midgut, salivary gland, testis, and ovary) by using the RNA Simple Total RNA Kit (TIANGEN Biotech, Beijing, China) following the recommended protocol. All these tissues were collected from the adult stage and used to extract RNA. The extracted RNA was treated with DNAase I (TIANGEN Biotech, Beijing, China) following its protocol.

The cDNA was prepared using 1 μg extracted RNA as a template following the protocol of the One-step RT-PCR kit (Takara Biomedical Technology, Beijing, China). The qPCR reaction mixtures were prepared by using the GoTaq^®^ qPCR master mix (Promega(Madison, WI, USA)) and gene-specific primers (Table S1). The qPCR reactions were carried out under the following conditions: 94 °C for 3 min; 94 °C for 40 s, and 62 °C for 1 min for 35 cycles. The relative expressions of targeted genes were normalized with the endogenous reference gene, ribosomal protein L32 (*PxRPL32*) [38]. Three biological replications and three technical replications were used for each gene. The relative gene expressions were calculated by using the 2^Δ^Ct method. All primers used in this study are listed in the Supplementary Table S1.

### 2.10. PCR Amplification and Cloning of PxCyc B1 Gene

The full-length coding sequence of the *PxCyc B1* gene was obtained from the DBM genomic database [30]. The primers were designed based on this obtained sequence. The PCR reaction mix was prepared with the Super-Fidelity DNA Polymerase (Vazyme, Nanjing, China) by using the adult female cDNA as the template, and PCR was conducted by using the following conditions: 94 °C for 4 min; 38 cycles of 94 °C for 15 s, 60 °C for 45 s, 72 °C for 1 min; 72 °C for 10 min; and then 4 °C forever. The amplified PCR product was purified by using the Omega gel purification Kit (Omega, Doraville, GA, USA) following its protocol. The purified PCR product was cloned into the PJET1.2 blunt-end vector (Thermo Scientific, Waltham, MA, USA) following the standard protocol and confirmed through sequencing.

### 2.11. Double-Stranded RNA Synthesis, Microinjections, and RNAi Analysis

To target the *PxCyc B1* gene using RNAi, a 540-bp DNA fragment based on the confirmed sequence was selected and used to design the primers (Table S1). The PCR reaction was performed with the following conditions: 94 °C for 4 min; 32 cycles of 94 °C for 45 s, 58 °C for 45 s, 72 °C for 30 s; 72 °C for 10 min, and then 4 ℃ forever. Furthermore, forward and reverse primers were designed with the T7 promoter at the 5′ end of these two primers (Table S1). The PCR reaction was prepared and carried out under the same conditions as the above by using the amplified PCR product as the template. The PCR product was purified by using the Omega gel purification Kit. The purified PCR product was further used as the template for dsRNA synthesis by using T7 RiboMAX™ Express RNAi Kit (Promega) [39,40].

The synthesized dsRNA (dsPxCyc-B1) was injected into the last stage of the pupae by using the microinjector specified for pupal injections. The pupae injected with dsEGFP were used as the control. A total of 300 pupae were injected for each dsRNA. Out of these injected pupae, a total of 100 pupae were collected 0 h, 12 h, 24 h, 36 h, and 48 h after injection and stored at −80 °C. The collected pupae from each time point were used for RNA extraction. The extracted RNA was used to prepare the cDNA.

Further, RT-qPCR was performed to determine the relative expression at different time points. The adults from the remaining pupae were then used for phenotypic analysis, ovary dissection, and crossing with opposite-sex wild-type individuals. The eggs were collected 1 day, 2 days, and 3 days after mating and the hatching rate of collected eggs was also calculated.

### 2.12. Statistical Analysis

GRAPHPAD 8.02 was used to perform one-way analysis of variance. Significant differences between the relative expression amongst different developmental stages and tissues were calculated by using Tukey’s test at *p* < 0.05. Similarly, Tukey’s test was used to examine the significant differences among the gene expression of dsPxCyc B1 treated group and the control group after different time points of injections and egg laying capacity and hatching rate in the treated group and control group after the dsRNA injection. Graphical illustrations were prepared by using mean ± SEM of at least three biological replications.

## 3. Results

### 3.1. Identificationsand Subcellular Localization of Cyclin Genes in P. xylostella

To identify the cyclin genes in the *P. xylostella* genome, HMMER3 (http://hmmer.janelia.org/ accessed on 10 May 2022) was used to search the cyclin-like genes in the whole genome by using cyclin-C and cyclin-N domains in the Pfam database. Initially, we screened a total of 31 genes in the genome. After removing the redundant and duplicated genes, we finalized 21 unique genes (Table 1). These cyclin genes were renamed according to their genome annotation (Supplementary Table S2). The length of the *P. xylostella* cyclin proteins ranged between 150 aa and 614 aa, with an average of 372 aa. The smallest cyclin protein is *PxCyc rel4* (172 aa), while the largest protein is *PxCyc* *T2* (614 aa). The CDS length, theoretical isoelectric point, and molecular weight of cyclin-like proteins ranged from 519 to 1845 bp, 6.3 to 9.7, and 15.84 to 69.27 KDa, respectively. Detailed information related to the physicochemical properties of cyclin-like genes is listed in Table 1.

The subcellular localization prediction of cyclin proteins showed that most cyclin proteins (17 out 21) were localized in the nucleus. Two cyclin proteins *PxCyc rel3* and *PxCyc A2* were localized in the nucleus and cytoplasm, *PxCyc rel5* was localized in the cytoplasm and extracellular membrane, and *PxCyc H* was localized in the cytoplasm (Table 1).

### 3.2. Gene Structure and Conserved Domain

**Figure 1:**
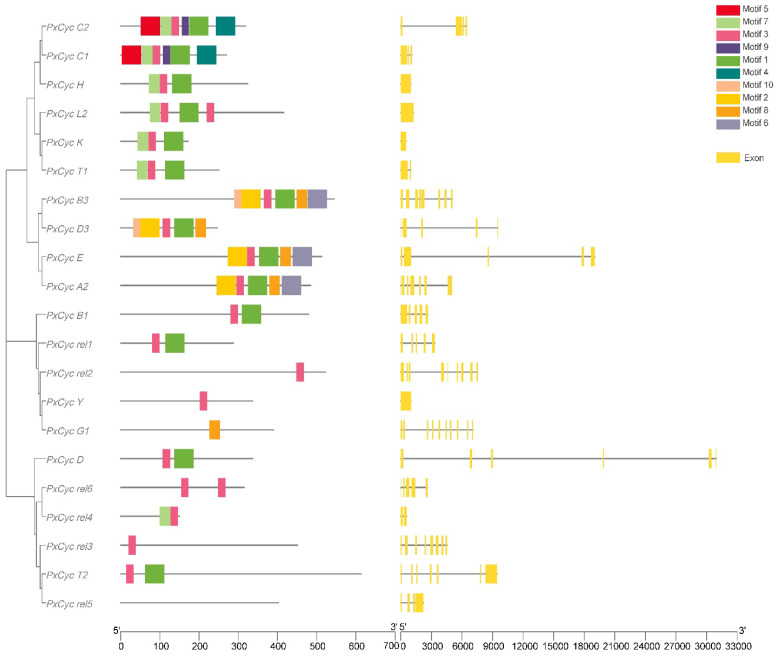
Conserved motif distribution patterns and gene structure (exons–introns distribution) of cyclin-like protein in *P. xylostella*. Commonly shared motifs among genes tend to cluster in the same groups, referring to their similar functions. Most cyclin-like genes are composed of one and five exons, and few genes are composed of the maximum number of exons (10).

Gene structure visualization showed that most cyclin-like genes of *P. xylostella* were composed of five exons (5 out of 21), followed by a single exon (4 out of 21), whereas the *PxCyc D* and *PxCyc G1* contained the maximum number of exons (10). Gene length variations of the cyclin gene of *P. xylostella* revealed that *PxCyc D3* possessed the longest genomic sequences of 9544 bp, while the *PxCyc rel4* had the smallest genomic sequence of 453 bp (Figure 1).

Many common and unique motifs in cyclin-like proteins were identified by scanning protein sequences at the MEME server. Genes with commonly shared motifs were clustered in the same groups, indicating their similar functions. Different motifs possessed different lengths. For example, motifs 1, 2, 4, 5, and 6 had a maximum length of 50 amino acid residues, while motif 10 possessed a minimum length of 18 amino acid residues. Motif 7 and motif 8 consisted of 28 amino residues, while motif 3 and motif 9 consisted of 20 and 19 amino acid residues, respectively (Supplementary Table S3). The conserved motif distribution patterns of cyclin-like proteins were shown in Figure 1.

The conserved domain analysis of cyclin genes in *P. xylostella* revealed that all 21 cyclin genes contained the cyclin N domain, 9 out of 21 cyclin genes contained both the cyclin N and cyclin C domain, while the remaining 11 cyclin genes only had the cyclin N domain. These results indicated that the cyclin N domain is highly conserved while the cyclin C domain is less conserved (Supplementary Figure S1).

### 3.3. Genomic Location and Phylogenetic Analysis

**Figure 2:**
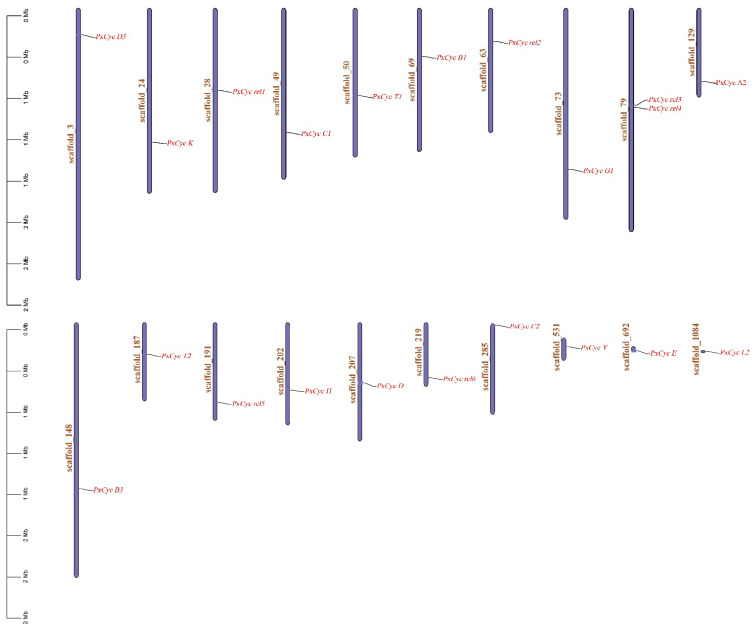
Genomic distribution of the *P. xylostella* cyclin-like proteins. Most of the scaffolds possessed a single cyclin-like protein except scaffold 79, which possessed two cyclin-like proteins.

Cyclin-like genes were unequally distributed from scaffold 3 to scaffold 1084 in the *P. xylostella* genome. The highest number of cyclin proteins were present on scaffold 79 (2 out of 21), followed by one cyclin-like protein present on scaffold 3, scaffold 24, scaffold 29, scaffold 49, scaffold 50, scaffold 63, scaffold 69, scaffold 129, scaffold 148, scaffold 187, scaffold 191, scaffold 202, scaffold 207, scaffold 219, scaffold 285, scaffold 531, scaffold 692, and scaffold 1082. The genomic distribution of all cyclin-like proteins is shown in Figure 2.

**Figure 3:**
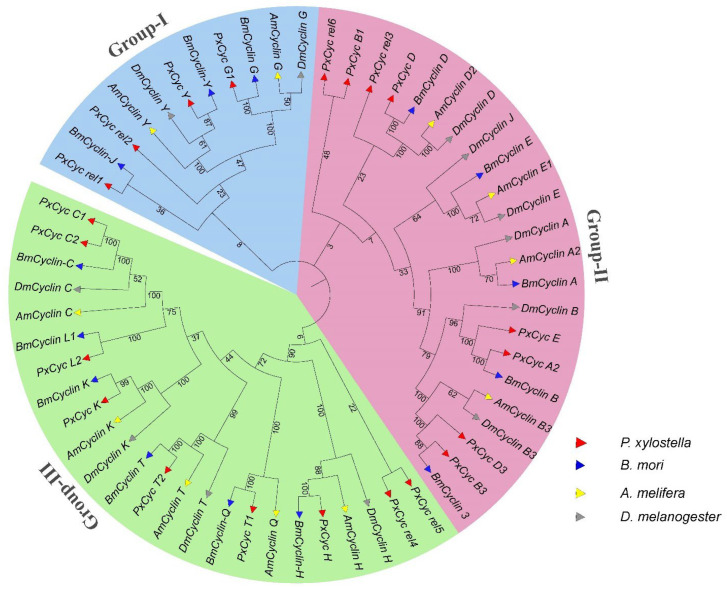
Phylogenetic relationships of the cyclin-like proteins of *P. xylostella* and three other insect species (*B. mori*, *D. melanogaster*, and *A. melefera*). The phylogenetic tree is classified into three groups. Different groups are highlighted with different colors. Group I, Group II, and Group III are highlighted with pink, light blue, and green colors, respectively.

The evolutionary relationships of the cyclin-like proteins of *P. xylostella* with three other insects (*B. mori*, *D. melanogaster*, and *Apis melifera*) from three different orders (Lepidoptera, Diptera, and Hymenoptera) were studied by constructing the phylogenetic tree. A total of 22, 34, and 14 genes were identified in *B. mori*, *D. melanogeter*, and *A. melifera*, respectively, which contained multiple isoforms for one gene. We finalized 14,12, and 11 genes of *B. mori*, *D. melanogeter*, and *A. melifera* for phylogenic analysis after removing redundant and duplicated sequences. Moreover, we removed multiple isoforms and used a single isoform to construct the phylogenetic tree (Supplementary Table S4). The phylogenetic relationships of cyclin-like genes were classified into three clades based on their bootstrap values. Group I showed the evolutionary relationships of *PxCyc rel1*, *PxCyc rel 2*, *PxCyc Y*, and *PxCyc G* with the other three insect species (Figure 3). The most important and conserved clade was Group II, which contained *PxCyc A2*, *PxCyc B1*, *PxCyc B3*, *PxCyc C1 PxCyc D3*, and *PxCyc E*, and the remaining cyclin genes were included in Group III (Figure 3).

### 3.4. Putative Cis-Regulatory Elements of Cyclin Genes

**Figure 4:**
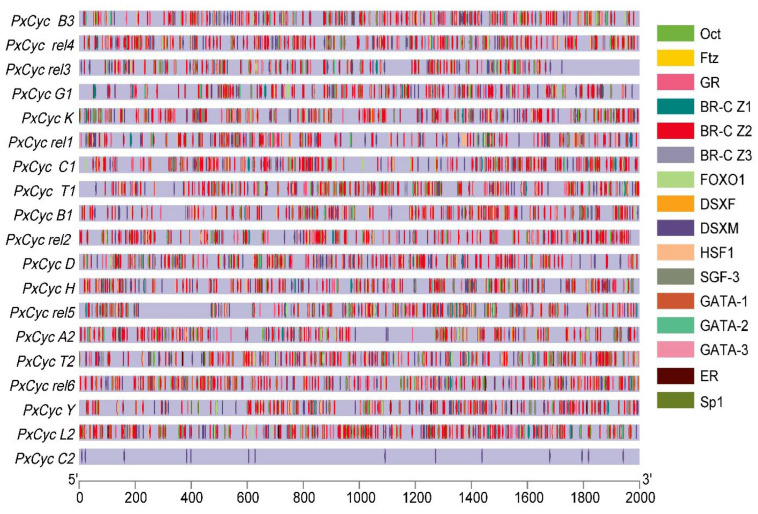
Schematic representation of the transcription factors present in the putative promoter region of cyclin genes of *P. xylostella*. Different elements are highlighted with different colors, and locations in the promoter are also marked with different colors.

*Cis*-regulatory elements play a vital role in the transcription of a gene in particular manners. The identification and prediction of *Cis*-regulatory elements provide a way to understand the role of a specific gene. To predict gene functions and their regulatory patterns, we analyzed the putative promoter region of cyclin genes. A large number of important transcription factors were predicted, except the core promoter elements such as TATA, CAAT, and GC box. These transcription binding elements were classified into different categories based on their functions, such as growth and development, reproduction, and sex-specific elements. Octamer binding factors (Oct); fushi tarazu factor (Ftz); glucocorticoid receptor binding sites; broad complex isoforms 1, 2, 3 (Br-C Z1, Br-C Z2, Br-C Z3); silk gland factor (SGF); forkhead box 1 (FOXO-1); GATA motif; and Sp1 binding sites were included in growth and development related elements. Estrogen receptor (ER) and male and female double sex binding sites (DSXM, DSXF) were included in reproductive and sex-specific related elements. All putative promoter sequences of cyclin genes showed enrichment of these transcription factors except for the *PxCyc C*, which possessed few transcription binding factors (Figure 4).

### 3.5. Identification of miRNA Targeting the Cyclin Genes

**Figure 5:**
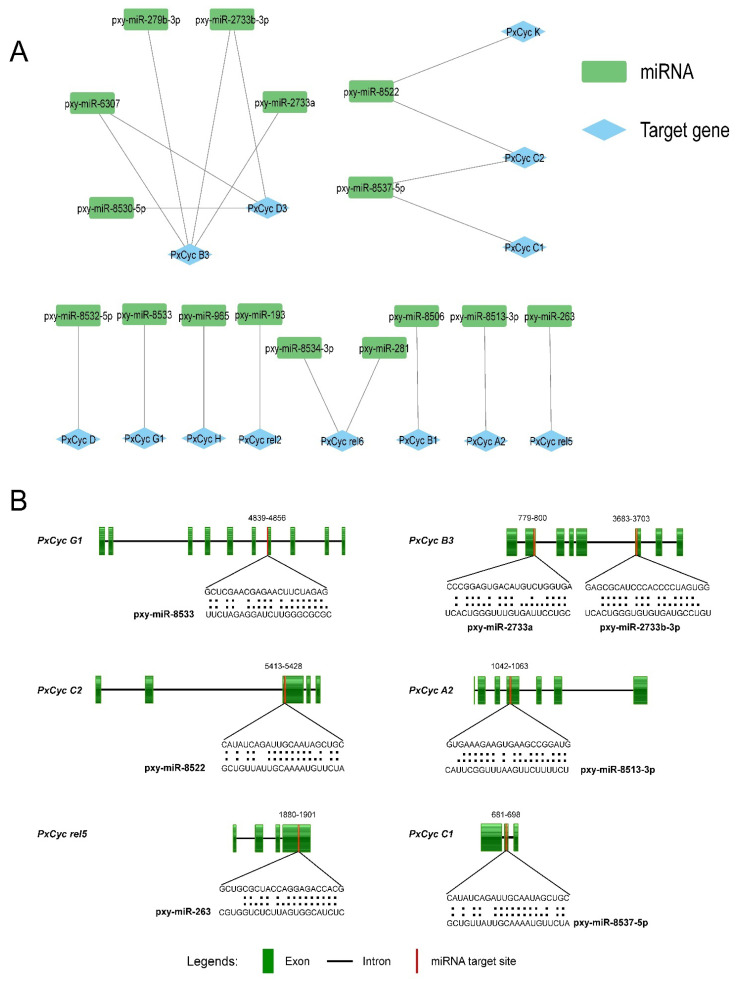
Predicted miRNAs targeting the cyclin genes of *P. xylostella*. (**A**), the network of 16 miRNAs targeting the 13 cyclin genes. (**B**), a schematic representation of the miRNA target sites in six cyclin genes. Green boxes represent the exon; black lines represent the introns; red lines represent the miRNA target sites. Upper sequences indicate the target sequences, and lower sequences indicate the miRNAs; black dots highlight the alignment of the target sequences with the miRNA sequences.

The miRNAs are small non-coding RNAs that regulate the gene expressions in animals and plants, primarily targeting the specific location inside the messenger RNA (mRNA) [41,42,43]. The miRNA prediction results indicated that 16 miRNAs were predicted to target the 13 cyclin genes of *P. xylostella* (Supplementary Table S5). Four miRNAs (pxy-miR-279b-3p, pxy-miR-2733a, pxy-miR-2733b-3p, pxy-miR-6307) targeted the *PxCyc B3*, while three miRNAs (pxy-miR-8530-5p, pxy-miR-2733b-3p, pxy-miR-6307) targeted the *PxCyc D3*. The *PxCyc C2* and *PxCyc rel6* genes were targeted by two miRNAs, respectively, while a single miRNA targeted the remaining nine cyclin genes. Four miRNAs (pxy-miR-8522, pxy-miR-2733b-3p, pxy-miR-6307, pxy-miR-8537-5p) targeted more than one cyclin gene (Figure 5A). The target sites of six cyclin genes along their miRNAs sequence and alignments are shown in Figure 5B. The functional validation of these predicted miRNAs and their role in gene regulation in *P. xylostella* will be key for future research directions.

### 3.6. Transcriptome-Based Expression Profiling of Cyclin-like Genes in P. xylostella

**Figure 6:**
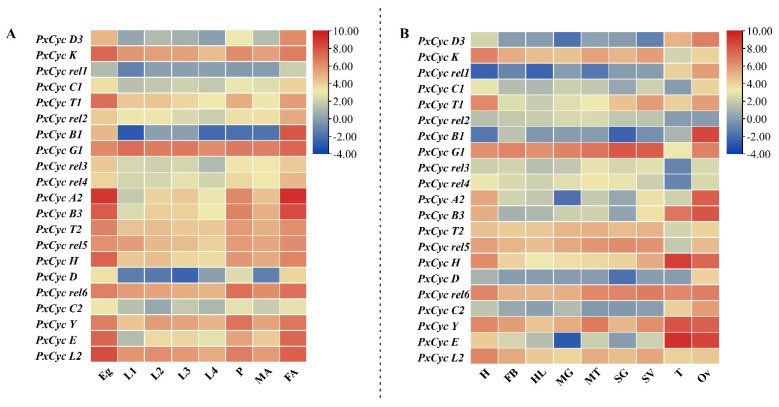
Heat map of transcriptomic expression profiling of cyclin genes at various stages and tissues. (**A**), the expression profile at various stages; (**B**), the expression profile of cyclin genes in different tissues. Log 2 transformed FPKM values were used for the heatmap construction. Eg, egg; L1, first-instar larva; L2, second-instar larva; L3, third-instar larva; L4-F, fourth-instar female larva; L4-M, fourth-instar male larva; P-F, female pupa; P-M, male pupa; A-M, adult male; A-F, adult female; HD, head; FB, fat body; HL, hemolymph; MG, midgut; MT; malpighian tubule; SG, silk gland; SV, salivary gland; TE, testis; OV, ovary.

The transcriptome-based data were used to calculate the expression patterns of 21 cyclin-like genes at different developmental stages, such as eggs, first-instar larvae, second-instar larvae, third-instar larvae, fourth-instar larvae, pupae, female adults, and male adults. Most cyclin-like genes, i.e., *PxCyc K*, *PxCyc T1*, *PxCyc A2*, *PxCyc B3*, *PxCyc T2*, *PxCyc H*, *PxCyc Y*, *PxCyc E*, and *PxCyc L2*, were highly expressed at the egg and adult female stages. Other genes (*PxCyc rel1*, *PxCyc C1*, *PxCyc rel2 PxCyc rel3*, *PxCyc rel4 PxCyc rel5*, *PxCyc H*, *PxCyc D*, *PxCyc rel6*, and *PxCyc C2*) were expressed almost at every developmental stage. One gene, *PxCyc B1*, was only expressed in adult females with little expressions at the egg stage (Figure 6A).

Furthermore, we evaluated the transcriptome-based expression profiling of 21 cyclin-like genes in different tissues such as the head, fat body, hemolymph, midgut, silk gland, salivary gland, ovary, and testis. Most cyclin genes (16 out of 21) were highly expressed in reproductive-related tissues such as the ovary and testis. Three cyclin genes (*PxCyc K*, *PxCyc T1*, and *PxCyc L2*) were highly expressed in the head, and *PxCyc rel5* was highly expressed in the silk gland. Surprisingly, *PxCyc B1* was only expressed in the ovary with no expression in other tissues (Figure 6B). Detailed information about the transcriptome-based expression values at different stages and tissues is listed in Supplementary Table S6. These results indicated that cyclin-like genes might have some distinct roles in the reproduction of *P. xylostella* rather than growth and development.

### 3.7. Validation of Cyclin Gene Expressions through RT-qPCR

**Figure 7:**
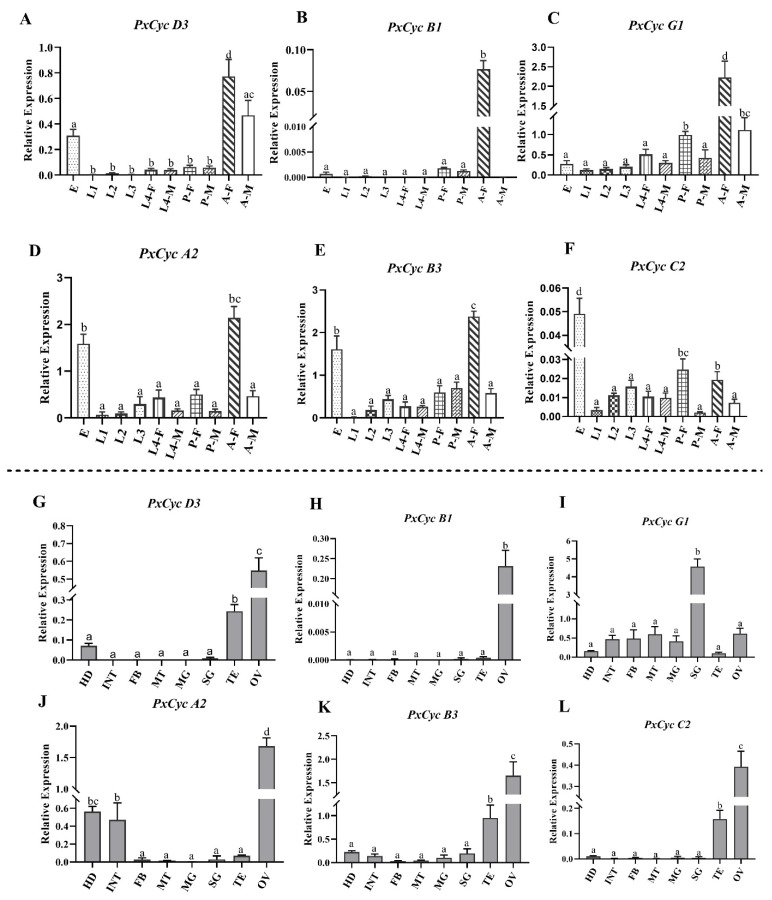
RT-qPCR-based validation of cyclin genes at various stages and tissues. (**A**–**F**), relative expression of six cyclin-like genes at different stages; (**G**–**L**), expression of six cyclin-like genes at different tissues. Bars represent the mean ± SEM of three biological replications; different letters highlight the significant difference in relative expressions among the different stages and tissues. Eg, egg; L1, first-instar larva; L2, second-instar larva; L3, third-instar larva; L4-F, fourth-instar female larva; L4-M, fourth-instar male larva; P-F, female pupa; P-M, male pupa; A-M, adult male; A-F, adult female; HD, head; INT, integument; FB, fat body; MG, midgut; MT, malpighian tubule; SG, silk gland; TE, testis; OV, ovary.

RT-qPCR was performed to confirm transcriptomic expression profilings of cyclin-like genes at different stages and tissues. Based on previous reports in *B. mori* and *D. melanogaster* [12,15,16,19], six (*PxCyc D3*, *PxCyc B1*, *PxCyc G1*, *PxCyc A2*, *PxCyc B3*, *PxCyc D*, *PxCyc C2*) genes were selected. *PxCyc D3* and *PxCyc G1* exhibited significantly high relative expressions in adult females, followed by adult males and eggs. Similarly, *PxCyc A2* and *PxCyc B3* also showed significantly high relative expressions in the adult, followed by egg. The relative expressions of the *PxCyc B1* were only detected in the adult female with very few expressions in other stages. Interestingly, the *PxCyc C2* showed significantly high relative expressions at the eggs, followed by female pupae and female adults (Figure 7A–F).

Next, we performed RT-qPCR for six cyclin genes to confirm their relative expressions in different tissues. *PxCyc D3*, *PxCyc B3*, and *PxCyc C2* showed significantly high relative expression in the ovary, followed by the testis. The *PxCyc A2* exhibited significantly high relative expression at the ovary, followed by head and integument. The relative expression of *PxCyc B1* was only detected in the ovary (Figure 7G–L). These results were somehow consistent with transcriptome expression data, which indicated that the cyclin-like genes might play some roles in reproduction along with growth and development.

### 3.8. RNAi of PxCyc B1 Gene

**Figure 8:**
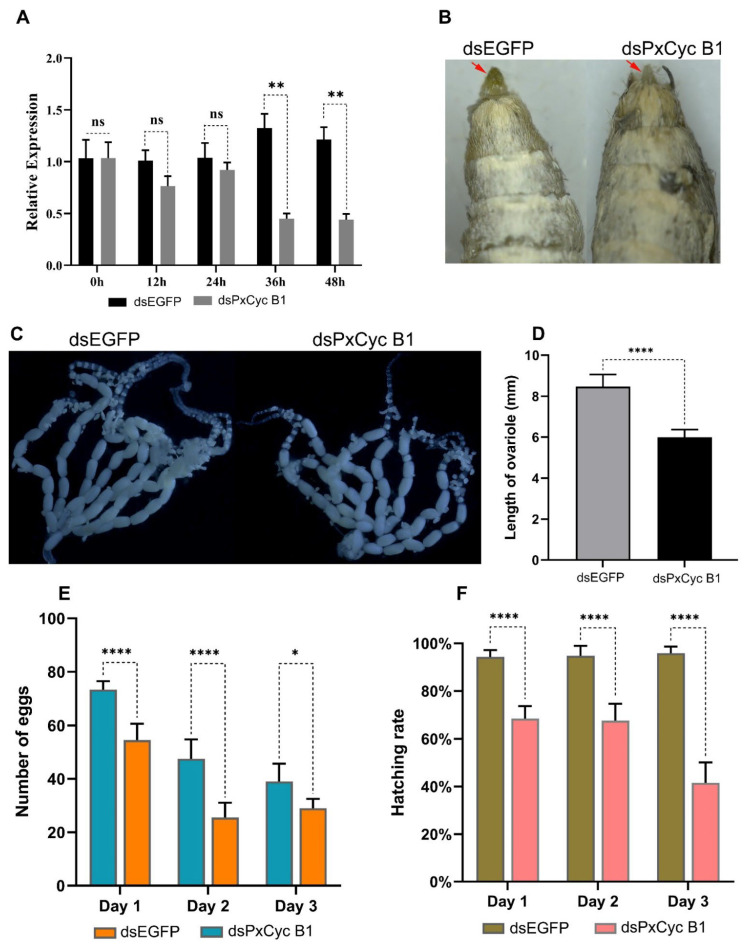
RNAi analysis of *PxCyc B1*. (**A**), relative expressions of *PxCyc B1* at different time points after dsRNA injections; (**B**), phenotypic differences in external genitalia after dsRNA injections; (**C**), differences in ovary between dsPxCyc B1 and dsEGFP, (**D**), length of ovariole; (**E**), the difference in the number of eggs laid by females injected with dsPxCyc B1 and dsEGFP, (**F**), the difference in hatching rate. Bars represent the mean ± SEM of at least three biological replications. Significant differences were highlighted with asterisks; **** indicates the highly significant difference at *p* < 0.001; ** indicates the significant difference at *p* < 0.01; * indicates the significant difference at *p* < 0.05; and ns for the non-significant difference.

Based on RT-qPCR results, we selected one gene *PxCyc B1* for downstream functional validation through RNAi because *PxCyc B1* was only expressed in the ovary tissue of adult females and may play specific functions in the ovary development and reproduction of *P. xylostella*. For this purpose, the full-length 1440 bp coding sequence of *PxCyc B1* was PCR amplified and cloned (Supplementary Figure S2A). A dsRNA of 502 bp sequence was designed to target the conserved region of *PxCyc B1* (Supplementary Figure S2B). A total of 300 pupae for each dsRNA (*PxCyc B1* and EGFP) were injected. Out of these injected pupae, 200 pupae survived with a survival rate of 67%. The injected pupae were used to detect the relative expressions of *PxCyc B3* at different time points (0 h, 12 h, 24 h, 36 h, and 48 h) after injections, which indicated that relative expressions of *PxCyc B1* were significantly downregulated compared with the control 36 h and 48 h after injections (Figure 8A).

Next, we explored whether the suppression *PxCyc B1* caused any phenotypic defects in females. The injected females were observed under a stereoscope and all female body parts were found to be normal except the external genitalia. The external genitalia of *dsPxCyc B1*-injected female were smaller than that of the wild-type female (Figure 8B).

To determine whether the suppression of *PxCyc B1* caused any defect in ovary development, we dissected 20 dsPxCyc B1-injected females and observed them under the microscope (Figure 8C). Overall, the ovary was normal, but the length of the ovariole was significantly smaller than that of the wild type (Figure 8D).

Finally, we observed the egg-laying capacity and hatching rate of dsPxCyc B1-injected females for up to three consecutive days. The results exhibited that the egg-laying capacity and hatching rate were significantly lower in the females with dsPxCyc B1 injections than in the females with dsEGFP injections (Figure 8E,F). These findings demonstrated that dsPxCyc B1 plays a significant role in female reproductive development and female fertility.

## 4. Discussion

The combination of cyclins and CDKs is essential for cell division during the cell cycle progression [2,44]. The majority of cyclin genes participate in nearly all of mitosis through interactions with CDKs and other proteins, and they are essential for the growth and development of animals, plants, and insects [45,46,47]. The fast advancement of genome sequencing and bioinformatics has enhanced the availability of entire sets of cyclin genes from a variety of species, including *Arabidopsis* [48], *Oryza sativa* [49], *Zea mays* [50], tomato [51], *Caenorhabditis elegans* [52], yeast [52], and human [53]. Comprehensive identification and characterization of the cyclin gene might open the way for a more in-depth functional investigation of these proteins. However, gene coding for cyclin proteins have not been well explored in insects and even less in *P. xylostella*. To date, few cyclin genes have individually been characterized in insects, but no genome-wide identification and characterization of a complete set of cyclin genes were studied.

Previous studies on genome annotation reported 31 genes in the *P. xylostella* genome that were annotated as cyclin-like genes [25]. However, we identified 21 unique cyclin genes in the *P. xylostella* genome after removing redundant and duplicated genes (Table 1). Moreover, a few genes in *P. xylostella* were annotated as cyclin-like genes but did not contain a cyclin core domain and were considered to remove these genes. The number of cyclin genes varies across the species, such as 52 genes in *Arabidopsis* [48], 22 in human [53], 34 in *Caenorhabditis elegans* [52], 59 in *Zea mays* [50], and 23 in yeast [52]. The number of genes in a species belonging to the same group/family depends on genome size and complexity. Cyclin genes homologous in insects are fewer than the plant species mainly because of small genome size and fewer chromosomes. Toward the subcellular localization of cyclin genes, we discovered that the majority of cyclins (85.90%) were localized in the nucleus (Table 1), which might be because the cyclin genes interact with CDKs and regulate the cell cycle process [54]. Furthermore, the cyclin genes primarily involve cell division during mitosis, which might be the reason for the nucleus localization of most cyclin genes [55]. The evolutionary process has resulted in the structural diversity of gene families and the number of exons and introns that play a significant role in gene expressions. Exons counted in cyclin genes ranged from 1 to 10 (Figure 1). However, most cyclin genes comprised five exons (5 out of 21) and a single exon (4 out of 21). A single exon in most cyclin genes may be due to intron deletions during the evolutionary process. Most cyclin genes share common motifs, such as motif 1, motif 5, and motif 10 (Figure 1), which indicates that the gene that shares the same motif may perform the same functions. However, further studies are required to understand the variations in gene structure and functions of the same motif present in different cyclin genes. Additionally, with structure and motif, we found that the cyclin N domain is highly conserved and present in all cyclin genes, while cyclin C is less conserved. These results are similar to previous studies in other species; the cyclin N domain is highly conserved, which may be required for functions of these cyclin genes [48,49]. The phylogenetic relationship of *P. xylostella* cyclin genes with three other species indicates that most *P. xylostella* cyclin genes are closely related to the *B. mori* cyclin gene. This might be because the *P. xylostella* and *B. mori* belong to the same insect order (Lepidoptera). Notably, *D. melanogaster*, *B. mori*, and *A. melifera* possess fewer genes than *P. xylostella*, indicating that *P. xylostella* gained more cyclin genes during evolution. On the other hand, *D. melanogaster*, *B. mori*, and *A. melifera* might have lost some genes during the evolutionary phase [30]. This evolutionary status is well supported by molecular phylogeny of the Lepidopteran order, indicating the importance of *P. xylostella* in the history of lepidopteran evolution. The literature showed that the *P. xylostella* genome possesses a relatively larger set of genes and a moderate number of gene families, indicating the expansion of certain gene families in *P. xylostella* [30].

The putative *cis*-regulatory elements of a specific gene are essential for controlling the gene expression in stage-, tissue- and sex-specific manners [56]. In this study, we found different types of binding factors in the 2kb upstream putative promoter sequences of all cyclin genes (Figure 4). These identified factors are essential for insect growth and development, reproduction, and sex differentiation. The Oct binding factors belong to POU transcription factors, which involve cell differentiation of the nervous system and other organs during embryogenesis [55]; Ftz transcription factor regulates the cuticular protein and plays an essential role in the leg development of *Culex pipiens* [57]. Br-C Z isoforms encode a family of transcription factors that play a critical role in metamorphic processes and are a primary responder gene in the ecdysteroid signaling pathway [58]. These transcription factors have distinct functions in larvae and pupae development in different insect species [58,59,60,61]. SGF transcription factor involves silk gland formation; FOXO-1 binding factor regulates juvenile hormone degradation and controls the growth and development of *B. mori* [62]. ER and other receptors control the reproduction of insects [61]; DSXF and DSXM are two isoforms of the doublesex gene that have distinct roles in sex differentiation [26]. Next, we identified 16 miRNAs targeting the 13 different cyclin genes of *P. xylostella* (Supplementary Table S5). Out of these identified miRNAs, miR-279 is expressed in almost all developmental stages and plays a significant role in the entire life of *Aedes agypti* [63]; miR-281 is highly expressed in the midgut of *Ae. albopictus*, which enhances the viral replication [64]; miR-279-3p targets the *CYP325BB1* gene and regulates the deltamethrin resistance in *Culex pipiens* [65]; miR-263 and miR-263 regulate the chitin synthesis genes to control the molting and metabolism of locusts [66]. These studies indicate that predicted miRNAs targeting the cyclin genes may play crucial roles in regulating the genes important for the growth and development of *P. xylostella*.

Transcriptome-based expression profiling of cyclin genes revealed that most cyclin genes are highly expressed in reproductive tissues of adult insects except a few cyclin genes, which showed high expressions in other stages and tissues (Figure 6). Furthermore, RT-qPCR of six cyclin genes was performed, indicating the somehow consistency of our transcriptome data. High expressions of cyclin genes in reproductive tissues indicate that cyclin genes may have some distinct role in reproduction beyond the growth and development. Previous studies in some model organisms show that cyclin genes play specific roles in ovary development and oogenesis [67,68]. In *D. melanogaster*, cyclin B and cyclin B3 are involved in female and male fertility [15], while cyclin J and cyclin G are involved in egg chamber maturation and wing development [16,68]. In *B. mori*, cyclin B and B3 are involved in cell cycle completion in the BmN cell line [19]. The functions of each cyclin gene vary across the species. However, additional research is necessary to determine the probable function of each cyclin gene in *P. xylostella*.

Next, we performed RNAi-mediated suppression of *PxCyc B1*, which is only expressed in the ovary of adult females. Suppression of *PxCyc B1* disrupted external genitalia and decreased the length of the ovariole in female adults. Furthermore, the egg-laying capacity and hatching rate were also significantly decreased by suppressing the *PxCyc B1*, indicating the role of cyclin genes in the reproduction and fertility of *P. xylostella* (Figure 8). These results are consistent with previous studies in *D. melanogaster*, in which both male and female fertility were influenced by B-type cyclins [16]. However, in *B. mori*, B-type cyclins are not related to fertility but are involved in the progression of the cell cycle [19]. Moreover, cyclin B1 is involved in mouse reproduction, which has a specific role in oocyte maturation [69]. Considering previous studies, we concluded that the B-type cyclin genes are important for reproduction, fertility, and cell cycle regulation in different species, including insects. Furthermore, the *PxCyc B1* gene might be used as a target site for the development of an effective way to manage *P. xylostella* [70].

## 5. Conclusions

In conclusion, we identified 21 cyclin genes in the genome of *P. xylostella* through a comprehensive genome-wide analysis and evaluated the gene structure, genomic location, and evolutionary relationship. *Cis*-regulatory elements and potential miRNA targeting the cyclin genes were also assessed. By analyzing the transcriptomic and RT-qPCR-based expression profiling at different stages and tissues, we found that the majority of the cyclin genes were significantly expressed in the reproductive tissues of *P. xylostella*. Moreover, RNAi-mediated characterization of *PxCyc B1* showed its role in female fertility. Collectively, the current study provides the basis to identify the potential role of cyclin genes in the reproduction of insects, and the cyclin genes may be considered an effective target site to control the pest population.

## Figures and Tables

**Table 1:** Cyclin-like protein members in *P. xylostella* and their physicochemical properties.

Gene ID	Name	Position	Gene (bp)	CDS (bp)	Exon	Protein aa	MW (KDa)	PI	Subcellular Localization
Px008345	*PxCyc D3*	scaffold_3:189765..199308 −	9544	744	5	247	28.86152	6.9	Nucleus
Px006817	*PxCyc K*	scaffold_24:973210..973728 +	519	519	1	172	69.27571	9.0	Nucleus
Px007867	*PxCyc rel1*	scaffold_28:594896..598229 −	3334	867	5	288	61.94629	9.0	Nucleus
Px012463	*PxCyc C1*	scaffold_49:902075..903146 +	1072	813	3	270	59.41365	6.5	Nucleus.
Px012660	*PxCyc T1*	scaffold_50:635009..635991 +	983	756	2	251	57.4899	6.6	Nucleus.
Px014457	*PxCyc rel2*	scaffold_63:235869..243413 −	7545	1569	9	522	54.52173	9.6	Nucleus
Px015088	*PxCyc B1*	scaffold_69:348318..350973 −	2656	1440	5	479	54.46445	9.7	Nucleus
Px015665	*PxCyc G1*	scaffold_73:1165490..1172567 +	7078	1173	10	390	48.74312	5.8	Nucleus
Px016300	*PxCyc rel3*	scaffold_79:702120..706664 −	4545	1356	8	451	45.15651	6.3	Cytoplasm/Nucleus
Px016302	*PxCyc rel4*	scaffold_79:719945..720507 −	563	453	2	150	43.66896	8.1	Nucleus
Px001947	*PxCyc A2*	scaffold_129:528131..533128 +	4998	1458	7	485	38.2414	9.0	Cytoplasm/Nucleus
Px003066	*PxCyc B3*	scaffold_148:1207690..1212770 −	5081	1638	8	545	37.60928	9.2	Nucleus
Px004935	*PxCyc T2*	scaffold_187:227985..237433 −	9449	1845	7	614	37.60928	9.7	Nucleus
Px005179	*PxCyc rel5*	scaffold_191:581647..583871 +	2225	1209	4	402	37.5119	5.0	Cytoplasm/Extracell
Px005660	*PxCyc H*	scaffold_202:491765..492739 −	975	975	1	324	37.30218	6.8	Cytoplasm
Px005787	*PxCyc D*	scaffold_207:424017..454987 +	30,971	1014	6	337	34.33513	6.5	Nucleus
Px006186	*PxCyc rel6*	scaffold_219:400848..403467 +	30,971	948	5	315	31.59559	8.6	Nucleus
Px008001	*PxCyc C2*	scaffold_285:6758..13234 −	6477	957	4	318	31.13283	7.6	Nucleus
Px013211	*PxCyc Y*	scaffold_531:63257..64267 −	1011	1011	1	336	28.2765	5.6	Nucleus
Px015145	*PxCyc E*	scaffold_692:22981..42013 +	19,033	1542	5	513	20.30056	5.0	Nucleus
Px000785	*PxCyc L2*	scaffold_1084:7808..9058 +	1251	1251	1	416	15.84307	9.8	Nucleus

Note: MW = molecular weight, PI = theoretical isoelectric point.

## Data Availability

Not applicable.

## Supplementary Materials

The following supporting information can be downloaded at https://www.mdpi.com/article/10.3390/biology11101493/s1. Figure S1: Schematic representation of conserved domains in cyclin genes; Figure S2: Gel electrophoresis images for PCR amplification of *PxCyc B1* and synthesized dsRNAs; Table S1: Primer used in this study; Table S2: Protein sequences of cyclin genes of *P. xylostella*; Table S3: Conserved motif details; Table S4: Details and sequences of three different species used for phylogenetic analysis; Table S5: Details of identified miRNAs targeting the different cyclin genes of *P. xylostella*; Table S6: Details of transcriptomic data used to construct the heatmap.
