# Supplementary material for: Genome-Wide Identification, Expression Profiling, and Characterization of Cyclin-like Genes Reveal Their Role in the Fertility of the Diamondback Moth
Source: Biology (Basel). 2022 Oct 12;11(10):1493. doi: 10.3390/biology11101493 (PMC9598266; doi:10.3390/biology11101493)
Supplement: Supplementary file 1 [file biology-11-01493-s001.zip › SI Figures.pdf]

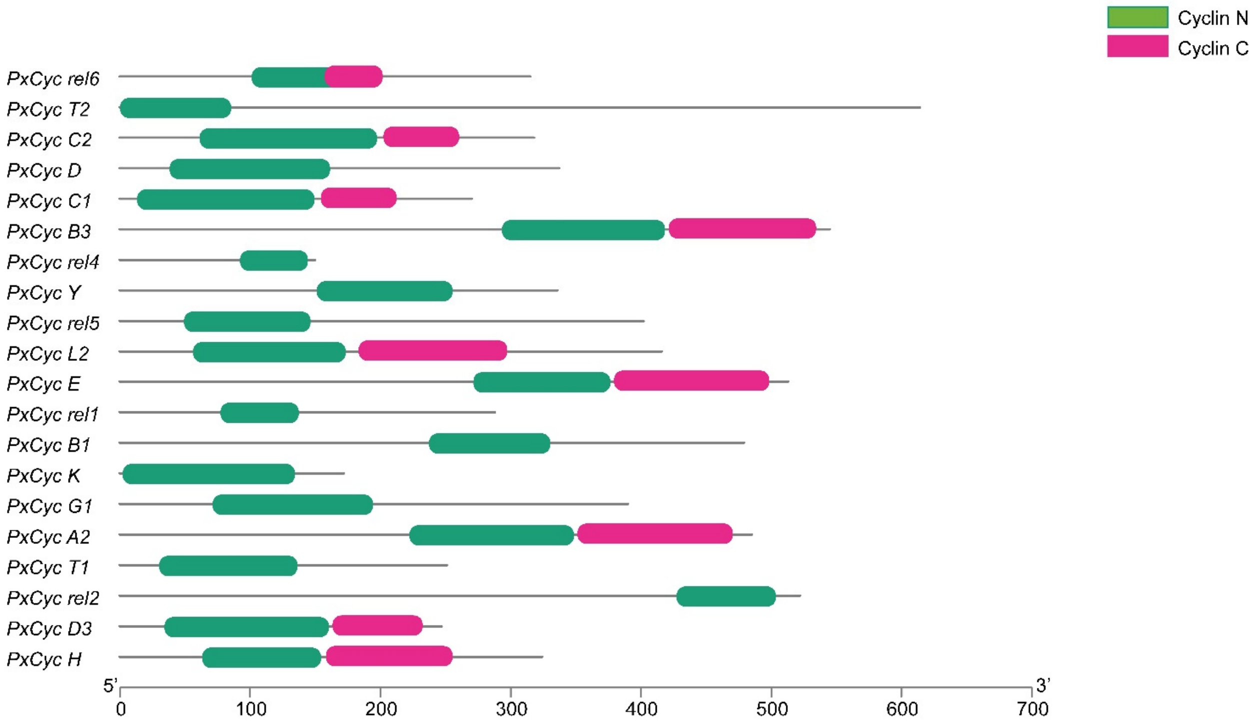

Figure S1: Schematic representation of conserved domains in cyclin genes. Two conserved domains cyclin N and cyclin are highlighted with different colors.

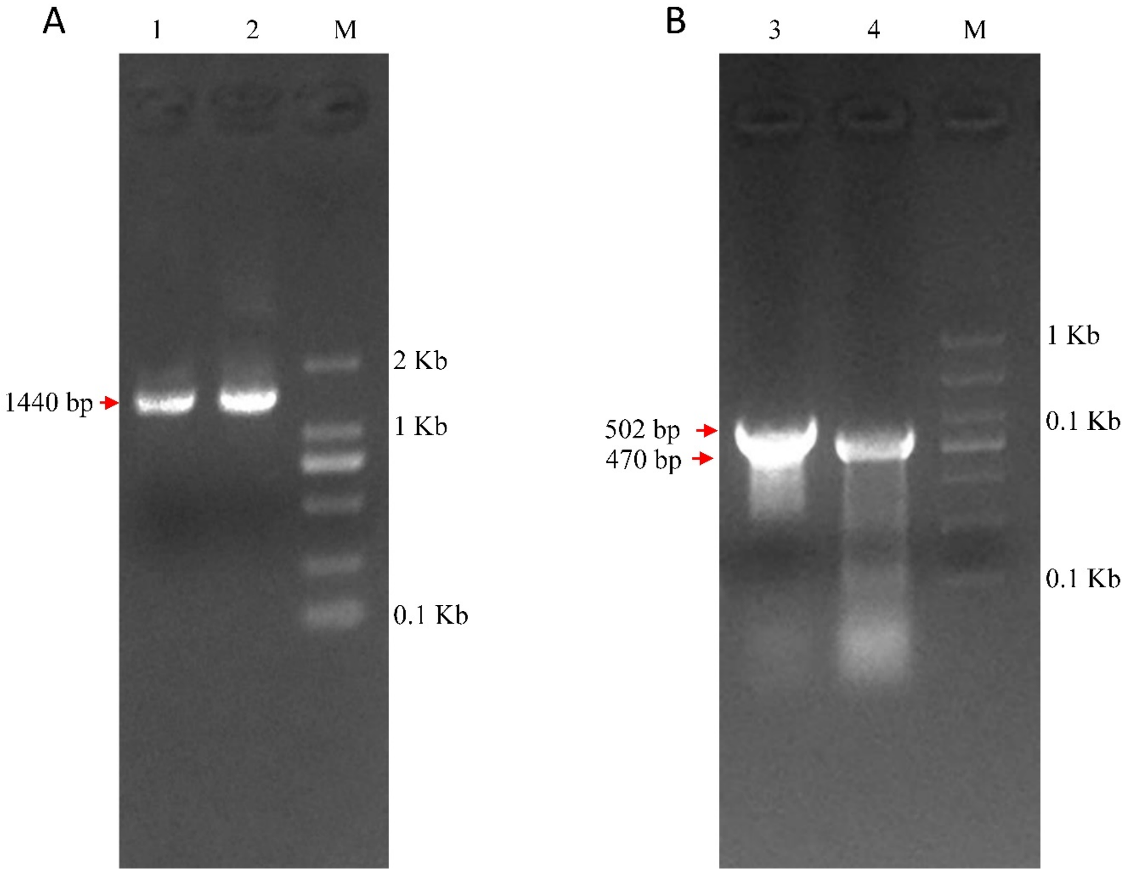

Figure S2: Gel electrophoreses images for PCR amplification of *PxCyc B1* and synthesized dsRNAs. A; gel electrophoreses image of PCR amplification of *PxCyc B1* gene, B; gel electrophoreses image of synthesized dsRNAs. Lane M; marker, Lane 1,2; PCR amplification of *PxCyc B1*, Lane 3; synthesized dsRNA of *PxCyc B1* and Lane 4; synthesized dsRNA of EGFP.
