# Supplementary material for: Genome-Wide Identification, Expression Profiling, and Characterization of Cyclin-like Genes Reveal Their Role in the Fertility of the Diamondback Moth
Source: Biology (Basel). 2022 Oct 12;11(10):1493. doi: 10.3390/biology11101493 (PMC9598266; doi:10.3390/biology11101493)
Supplement: Supplementary file 1 [file biology-11-01493-s001.zip › Table S1.pdf]

SI Table S1: Primer used in this study

Name	Sequence 5' to 3'	Use
qPCR CycB3-F	CAACGAAGCAAGGGCATCAC	
qPCR CycD3-R	CGTCAAACTTAGCGGCAATA	
qPCR CycG1-F	TGAAACGCATGTCGTCCATC	
qPCR CycG1-R	CGATCTCCACCAGGTTCTCC	
qPCR CycA2-F	GAATCAACACTGAAACCCAT	
qPCR CycA2-R	TCTAGCTCAATCAGGTAGCG	RT-qPCR validation
qPCR CycB3-F	TCCAAATCGACGACTACCTG	
qPCR CycB3-R	TCAAACTTAGCGGCAATAAA	
qPCR CycC2-F	TGATTGTTTACCAGCCGTACCG	
qPCR CycC2-R	GACATCTGTCCTCAGGGAGT	
qPCR CycB1-F	TTGGGGTTGCTGCGTTTCC	
qPCR CycB1-R	AGCTCGATCTCGCCAGGGTG	
CycB3-F	ATACCGCTCCCGTTAGACC	Cloning of *PxCycB3*
CycB3-R	CTACAGATTGAACTCAGGCAC	
dsCycB1-F	TAATACGACTCACTATAGGTGCAAGGCCTGTCACCTATAT	
dsCycB1-R	TAATACGACTCACTATAGGGGAAACGCAGCAACCCCAAA	dsRNA synthesis
dsEGFP-F	TAATACGACTCACTATAGGGGCTTCTCGTTGGGGTCTTTG	
dsEGFP-R	TAATACGACTCACTATAGGGACCACATGAAGCAGCACGAC	

Note: Underline primer sequences represent the T7 promoter for dsRNA synthesis
